
JOURNAL INFORMATION
==============================
NLM Title Abbreviation: J Vet Intern Med
Journal ID: J Vet Intern Med
NLM Journal ID: 8708660
Journal ID: JVIM

Journal of Veterinary Internal Medicine

ISSN: 0891-6640
EISSN: 1939-1676
Publisher: Oxford University Press

ARTICLE INFORMATION
==============================
PMCID: PMC11474614
DOI: 10.1111/jvim.16462
Article ID: JVIM16462
Article version: 1
Subjects: Issue Information, Issue Information

Issue Information

Electronic publication date: 2023 Nov 19
Publication date: 2023 Nov-Dec
Volume: 37
Issue: 6
First page: 1947
Last page: 1956
Copyright: © 2023 The Authors. Journal of Veterinary Internal Medicine published by Wiley Periodicals LLC on behalf of American College of Veterinary Internal Medicine.
License: This is an open access article under the terms of the http://creativecommons.org/licenses/by/4.0/ License, which permits use, distribution and reproduction in any medium, provided the original work is properly cited.
License URL: https://creativecommons.org/licenses/by/4.0/

Figure count: 0
Table count: 0
Page count: 1

source-schema-version-number: 2.0
cover-date: November/December 2023
details-of-publishers-convertor: Converter:WILEY_ML3GV2_TO_JATSPMC version:6.4.9 mode:remove_FC converted:15.10.2024

==============================

Volume 37, Number 6

November/December 2023

### JOURNAL OF VETERINARY INTERNAL MEDICINE

Open Access

ACVIM

American College of Veterinary Internal Medicine

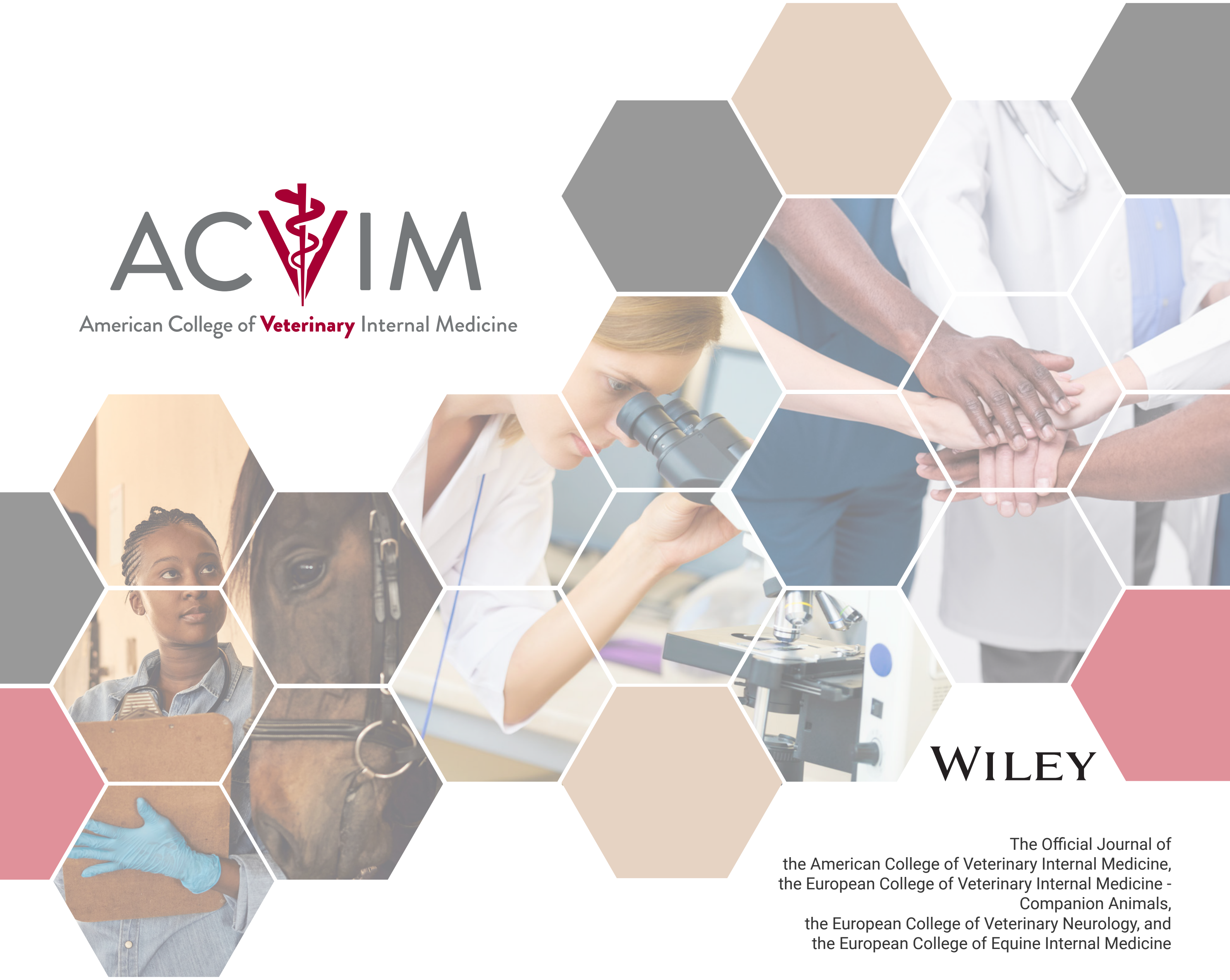

WILEY

The Official Journal of
the American College of Veterinary Internal Medicine,
the European College of Veterinary Internal Medicine - Companion Animals,
the European College of Veterinary Neurology, and
the European College of Equine Internal Medicine

### Journal of Veterinary Internal Medicine

Open Access

ACVIM American College of Veterinary Internal Medicine

The Official Journal of the American College of Veterinary Internal Medicine,
the European College of Veterinary Internal Medicine - Companion Animals, the European College of Veterinary Neurology,
and the European College of Equine Internal Medicine

**MISSION STATEMENT**

The mission of the *Journal of Veterinary Internal Medicine* is to advance veterinary medical knowledge and improve the lives of animals by publication of authoritative scientific articles of animal diseases.

**Volume 37, Number 6** **November/December 2023**

EDITORIAL

1957 **ACVIM-Endorsed Statements: Consensus statements, evidence-based practice guidelines and systematic reviews**
*ACVIM Consensus Statement Taskforce, Kenneth W. Hinchcliff, Paul S. Morley, Stephen P. DiBartola, Sandra D. Taylor, and Karyn A. Harrell*

CONSENSUS STATEMENT

1966 **Updated ACVIM consensus statement on leptospirosis in dogs**
*Jane E. Sykes, Thierry Francey, Simone Schuller, Robyn A. Stoddard, Larry D Cowgill, and George E. Moore*

SMALL ANIMAL

Standard Articles

*Cardiology*

1983 **Radiographic lung congestion scores in dogs with acute congestive heart failure caused by myxomatous mitral valve disease**
*Liza Koster, Jenna Vogel, Cary M. Springer, and Silke Hecht*

1992 **Localization and characterization of atrial depolarization waves on the surface electrocardiogram in dogs with rapid supraventricular tachycardia**
*Stefano Battaia, Manuela Perego, Damiano Cavallini, and Roberto Santilli*

2003 **Pharmacokinetics of pimobendan after oral administration to dogs with myxomatous mitral valve disease**
*Anna K. McManamey, Teresa C. DeFrancesco, Kathryn M. Meurs, and Mark G. Papich*

2011 **Use of serum osmolality to identify heart disease stage in dogs and relationship to mathematical chloride correction**
*Edward J. Daly, Autumn N. Harris, and Darcy B. Adin*

2021 **Utility of vertebral left atrial size and vertebral heart size to aid detection of congestive heart failure in dogs with respiratory signs**
*Evan S. Ross, Lance C. Visser, Nicholas Sbardellati, Brianna M. Potter, Alex Ohlendorf, and Brian A. Scansen*

2030 **Evaluation of right ventricular diastolic function, systolic function, and circulating galectin-3 concentrations in dogs with pulmonary stenosis**
*Randolph L. Winter, Kara L. Maneval, Claudio Serrano Ferrel, William A. Clark, Emily J. Herrold, and Jaylyn D. Rhinehart*

*Endocrinology*

2039 **Urine concentrating ability in cats with hyperthyroidism: Influence of radioiodine treatment, masked azotemia, and iatrogenic hypothyroidism**
*Mark E. Peterson and Mark Rishniw*

2052 **External validation of a United Kingdom primary-care Cushing's prediction tool in a population of referred Dutch dogs**
*Bart Eduardus Wilhelmus Ruijter, Céline Anne Bik, Imogen Schofield, and Stijn Johannes Maria Niessen*

2064 **Alterations in serum concentrations of visfatin and betatrophin in dogs with hypothyroidism**
*Michal Mazaki-Tovi, Omer Shachar, and Tzachi Even Zur*

2073 **A case-control survey study of environmental risk factors for primary hypoadrenocorticism in dogs**
*Amy E. Treeful, Kelly M. Searle, Dana M. Carroll, Kathleen J. Yost, Anna L. Hedger, and Steven G. Friedenberg*

*Gastroenterology*

2084 **Fuzapladib in a randomized controlled multicenter masked study in dogs with presumptive acute onset pancreatitis**
*Joerg M. Steiner, Chantal Lainesse, Yuya Noshiro, Yumiko Domen, Heather Sedlacek, Stephen E. Bienhoff, Kelly P. Doucette, David L. Bledsoe, and Hiroshi Shikama*

2093 **Pre-illness dietary risk factors in dogs with chronic enteropathy**
*Isla Trewin and Aarti Kathrani*

2102 **Long-term outcome of epileptic dogs treated with implantable vagus nerve stimulators**
*Thomas R. Harcourt-Brown and Michael Carter*

2109 **Effect of esomeprazole with and without a probiotic on fecal dysbiosis, intestinal inflammation, and fecal short-chain fatty acid concentrations in healthy dogs**
*Rae McAtee, Sarah M. Schmid, M. Katherine Tolbert, Scott Hetzel, Jan S. Suchodolski, and Jessica C. Pritchard*

2119 **Gastric pH and serum gastrin concentration in age-matched healthy dogs and dogs with chronic kidney disease**
*Kylie Grady, Eli Ernst, Patricia L. Secoura, Josh Price, Adam Birkenheuer, Shelly L. Vaden, Jonathan Lidbury, Emily Gould, Joerg M. Steiner, and M. Katherine Tolbert*

*General*

2125 **Evaluating the readability of recruitment materials in veterinary clinical research**
*Charly McKenna, Mindy Quigley, and Tracy L. Webb*

*Hematology*

2131 **Adverse effects of trazodone in dogs on primary hemostasis and electrocardiogram: A single-blinded placebo-controlled crossover study**
*Eduardo J. Benjamin, O. Lynne Nelson, Ryan Baumwart, and Jillian Haines*

2137 **Accuracy of Crit-Line® monitors in predicting hematocrit and change in blood volume of dogs during hemodialysis**
*Whitney E. Vickery, Sarah M. L. Steinbach, and George E. Moore*

2145 **No impact of polymorphism in the phosphodiesterase 5A gene in Cavalier King Charles Spaniels on pimobendan-induced inhibition of platelet aggregation response**
*Maria J. Reimann, Daniel N. Faisst, Mads Knold, Kathryn M. Meurs, Joshua A. Stern, Signe E. Cremer, Jacob E. Møller, Ingrid Ljungvall, Jens Häggström, and Lisbeth H. Olsen*

*Hepatology*

2157 **Prevalence and clinical relevance of cholelithiasis in cats: A multicenter retrospective study of 98 cases**
*Audrey Brunet, Cyril Duperrier-Simond, Suzanne Amoyal, Ghita Benchekroun, Juan Hernandez, Lorris Lecot, Thibaut Lurier, Jean-Luc Cadoré, and Emilie Krafft*

2171 **Occurrence and clinical relevance of postoperative hypernatremia in dogs undergoing cholecystectomy**
*Lara Brunori, Cormac Dolan, and Neus Elias Santo-Domingo*

2178 **Microbiome analysis of bile from apparently healthy cats and cats with suspected hepatobiliary disease**
*Tanner S. Slead, Benjamin J. Callahan, Megan E. Schreeg, Gabriela S. Seiler, Devorah M. Stowe, Maria Andrea Azcarate-Peril, Megan E. Jacob, and Jody L. Gookin*

*Nephrology/Urology*

2188 **Response and survival of dogs with proteinuria (UPC > 2.0) treated with angiotensin converting enzyme inhibitors**
*Emily A. Fulton, Alix R. McBrearty, Darren J. Shaw, and Alison E. Ridyard*

2200 **Adeno-associated virus-vectored erythropoietin gene therapy for anemia in cats with chronic kidney disease**
*Shelly L. Vaden, Allison R. Kendall, Jonathan D. Foster, Heidi L. New, Jane S. Eagleson, Jacky L. May, Anne M. Traas, Matthew J. Wilson, Beth H. McIntyre, Christian J. Hinderer, Lauren K. Olenick, and James M. Wilson*

2211 **Idiopathic functional urinary outflow tract obstruction in dogs, a retrospective case series (2010-2021): 31 cases**
*Kathrina Mathews, Christine Toedebusch, Carrie Palm, Allison Kendall, and Jodi L. Westropp*

2219 **Longitudinal examination of urine pH, specific gravity, protein, culture, and antimicrobial resistance profiles in healthy dogs**
*Andrew McGlynn, Ryan Mrofchak, Rushil Madan, Christopher Madden, Mohammad Jawad Jahid, Dixie Mollenkopf, Thomas Wittum, Sheryl S. Justice, Adam Rudinsky, Jessica Hokamp, and Vanessa Hale*

2230 **Population pharmacokinetic analysis of enrofloxacin and its active metabolite ciprofloxacin after intravenous injection to cats with reduced kidney function**
*Jonathan D. Foster, Mahmoud Abouraya, Mark G. Papich, and Nancy A. Muma*

2241 **Differentiation of stable kidney function versus progressive dysfunction in dogs**
*Larry D. Cowgill, Gilad Segev, Shelly Vaden, Sheri Ross, Cedric Dufayet, Leah A. Cohn, Mary Nabity, Giosi Farace, Donald Szlosek, Zenhwa Ouyang, Sarah Peterson, Melissa Beall, Murthy Yerramilli, and David Polzin*

2251 **Urinary cystatin B differentiates progressive versus stable IRIS Stage 1 chronic kidney disease in dogs**
*Gilad Segev, Shelly Vaden, Sheri Ross, Cedric Dufayet, Leah A. Cohn, Giosi Farace, Donald Szlosek, Zenhwa Ouyang, Sarah Peterson, Melissa Beall, Murthy Yerramilli, David Polzin, and Larry D. Cowgill*

2261 **Biological variation of urinary protein: Creatinine ratio and urine specific gravity in cats**
*Femke Mortier, Sylvie Daminet, Luc Duchateau, Ann Biscop, and Dominique Paepe*

*Neurology*

2269 **Risk factors, treatment, and outcome in dogs and cats with subdural hematoma and hemispheric collapse after ventriculoperitoneal shunting of congenital internal hydrocephalus**
*Daniela Farke, Anna K. Siwicka, Agnieszka Olszewska, Adriana Czerwik, Kathrin Büttner, and Martin J. Schmidt*

2278 **Relationship between magnetic resonance imaging findings and histological grade in spinal peripheral nerve sheath tumors in dogs**
*Simona Morabito, Swan Specchi, Pamela Di Donato, Danica Pollard, Ruth Dennis, Luisa De Risio, Nicholas J. Bacon, Maria Potamopoulou, Stefan Rupp, Daniele Corlazzoli, João Ribeiro, Francesca Cozzi, Konrad Jurina, Rodolfo Cappello, Edy Mercuriali, Katrin Beckmann, Thomas Flegel, Marika Menchetti, Florian König, Kaspar Matiasek, and Marco Rosati*

2291 **The efficacy and safety of cannabidiol as adjunct treatment for drug-resistant idiopathic epilepsy in 51 dogs: A double-blinded crossover study**
*Aaron J. Rozental, Brooke G. Weisbeck, Isabella Corsato Alvarenga, Daniel L. Gustafson, Breonna R. Kusick, Sangeeta Rao, Lisa R. Bartner, and Stephanie McGrath*

2301 **Idiopathic and structural episodic nonintentional head tremor in dogs: 100 cases (2004-2022)**
*Theofanis Liatis, Sofie F. M. Bhatti, Magdalena Dyrka, Rodrigo Gutierrez-Quintana, Rita Gonçalves, Megan Madden, and Steven De Decker*

2310 **Generalized myokymia, or neuromyotonia, or both in dogs with or without spinocerebellar ataxia**
*An Vanhaesebrouck, Mario Van Poucke, Kimberley Stee, Nicolas Granger, Edward Ives, Iris Van Soens, Ine Cornelis, Kenny Bossens, Luc Peelman, Luc Van Ham, and Sofie F. M. Bhatti*

*Nutrition*

2315 **Tolerability of naso-esophageal feeding tubes in dogs and cats at home: Retrospective review of 119 cases**
*Renaud Dumont, Julie Lemetayer, Loïc Desquilbet, and Elodie Darnis*

2322 **Efficacy of an elemental diet in achieving clinical remission in dogs with chronic enteropathy**
*Alison C. Manchester, Steven Dow, Lyndah Chow, Jason Gagne, and Michael R. Lappin*

2334 **Randomized controlled trial of hydrolyzed fish diets in dogs with chronic enteropathy**
*Kenneth W. Simpson, Meredith L. Miller, John P. Loftus, Mark Rishniw, Carol E. Frederick, and Joseph J. Wakshlag*

2344 **Safety of TCMCB07, a melanocortin-4 antagonist peptide, in dogs with naturally occurring cachexia**
*Sandra M. Axiak-Bechtel, Stacey B. Leach, Jessica R. Newton-Northup, Rowan J. Milner, Stacey A. Fox-Alvarez, Lana I. Fagman, Kaylee A. Young, Deborah J. Tate, Zachary M. Wright, John D. Chretin, Justin W. Allen, Sean K. Yoshimoto, Kimberly A. Selting, Brian K. Flesner, Carrie R. White, Tracy Mills, Michael Aherne, Philip J. Bergman, LeAnn Qi, Kenneth A. Gruber, and Michael F. Callahan*

*Oncology*

2356 **A retrospective study of sinonasal tumors in 182 dogs treated with stereotactic radiotherapy (3 × 10 Gy) (2010-2015)**
*Hiroto Yoshikawa, Mary H. Lafferty, Lynn R. Griffin, and Susan M. LaRue*

2368 **Long-term remission and survival in dogs with high-grade, B cell lymphoma treated with chemotherapy with or without sequential low-dose rate half-body irradiation**
*Matthew P. Best, Rod C. Straw, Elias Gumpel, and Darren R. Fry*

2375 **Recurrence and survival in dogs with excised colorectal polyps: A retrospective study of 58 cases**
*Tristan Méric, Julien Issard, Thomas Maufras, Marine Hugonnard, Odile Senecat, Alexis Lecoindre, Rodolfo Oliveira Leal, Coralie Bertolani, Olivier Toulza, Patrick Lecoindre, Elise Brisebard, Mireille Ledevin, Thibaut Larcher, Amandine Drut, Elodie Darnis, and Juan Hernandez*

2385 **Retrospective evaluation of ceruminous gland tumors confined to the external ear canal of dogs and cats treated with biopsy and $CO_2$ laser ablation**
*Jason B. Pieper, James O. Noxon, and Darren J. Berger*

2391 **Increased plasma DR-70 (fibrinogen-fibrin degradation products) concentrations as a diagnostic biomarker in dogs with neoplasms**
*Chiao-Hsu Ke, Ka-Mei Sio, Chun-Hung Wu, Yuan-Yuan Xia, Jih-Jong Lee, Chin-Hao Hu, Cheng-Chi Liu, Chueh-Ling Lu, Chiao-Lei Cheng, Keng-Hsuan Lin, Hirotaka Tomiyasu, Yu-Shan Wang, and Chen-Si Lin*

2402 **Individualized chemotherapy drug dose escalation in dogs with multicentric lymphoma**
*Jacob M. Siewert, Daniel L. Gustafson, Kristen M. Weishaar, Annie M. Galloway, and Douglas H. Thamm*

2410 **Novel genomic prognostic biomarkers for dogs with cancer**
*Esther Chon, Sharadha Sakthikumar, Min Tang, Matthew J. Hamilton, Andrew Vaughan, Ashley Smith, Breann Sommer, Cecilia Robat, Christina Manley, Christine Mullin, Emi Ohashi, Emily Manor, James Custis, Joanne Intile, Kai Biu Shiu, Lisa Parshley, Noelle Bergman, Sabina Sheppard-Olivares, Scott Hafeman, Zachary Wright, David Haworth, William Hendricks, and Guannan Wang*

*Pharmacology*

2422 **Pharmacokinetics and pharmacodynamics of intranasal and intramuscular administration of naloxone in working dogs administered fentanyl**
*Ciara A. Barr, Joanne Haughan, Giacomo Gianotti, Kelley Varner, Kenneth J. Drobatz, Darko Stefanovski, Mary Robinson, Mark Pennington, Amanda McGuire, and Cynthia M. Otto*

2429 **Oral cytarabine ocfosfate pharmacokinetics and assessment of leukocyte biomarkers in normal dogs**
*Danielle M. Zwueste, Karen M. Vernau, William Vernau, Bruno H. Pypendop, Heather K. Knych, Carlos A. Rodrigues, Amir Kol, Maria Questa, and Peter J. Dickinson*

*Respiratory*

2443 **Correlation of clinical and radiographic variables in cats with lower airway disease**
*Hannah Gareis, Lina Hörner-Schmid, Yury Zablotski, Jelena Palić, Silke Hecht, and Bianka Schulz*

2453 **Association between clinical history in the radiographic request and diagnostic accuracy of thorax radiographs in dogs: A retrospective case-control study**
*Natalie Arruda Bergamaschi, Lukas Huber, Eberhard Ludewig, Alexandra Böhler, Michaela Gumpenberger, Katharina M. Hittmair, Carina Strohmayer, Remco Folkertsma, and Conor Rowan*

2460 **Bronchial collapse and bronchial stenting in 9 dogs**
*Darren Kelly, Florence Juvet, Valerie Lamb, and Andrew Holdsworth*

2468 **Osteopontin and fibronectin in lung tissue, serum, and bronchoalveolar lavage fluid of dogs with idiopathic pulmonary fibrosis and control dogs**
*Aline Fastrès, Elodie Roels, Alexandru C. Tutunaru, Géraldine Bolen, Anne-Christine Merveille, Michael J. Day, Mutien-Marie Garigliany, Nadine Antoine, and Cécile Clercx*

Case Reports

*Endocrinology*

2478 **Wintersweet (*Chimonanthus praecox*) toxicity in 5 sheep**
*Cambrey Knapp, Thomas Van Dyke, and Derek Foster*

*Immunology*

2482 **Presumptive phenobarbital-induced systemic lupus erythematosus in a domestic dog**
*Erin Phillips, Matthew Kornya, Allison Collier, Maureen Barry, Katherine Morrison, and Felipe Reggeti*

*Nephrology/Urology*

2488 **Lesser vestibular periurethral gland-like inflammation associated with lower urinary tract signs in a female dog**
*Matthew Price, Tanner S. Slead, Patricia L. Secoura, Allison R. Kendall, and Shelly L. Vaden*

*Neurology*

2492 **Large T-cell extradural lymphoma with concurrent marked cerebrospinal fluid eosinophilia in a dog**
*Anna M. Massie, Katherine Skorupski, William Vernau, Ehren McLarty, Rachel V. Brady, and Karen M. Vernau*

2498 **Hereditary myotonia in cats associated with a new homozygous missense variant p.Ala331Pro in the muscle chloride channel ClC-1**
*Sílvia Corrêa, Roberta Martins Basso, Fabricio Moreira Cerri, José Paes de Oliveira-Filho, João Pessoa Araújo Jr, Sandra Regina Torelli, Livia Pinheiro Chagas da Cunha Salán, Maurício Oliveira Salán, Isabella Zeque Macedo, and Alexandre Secorun Borges*

2504 **Novel COL6A3 frameshift variant in American Staffordshire Terrier dogs with Ullrich-like congenital muscular dystrophy**
*Leanne Jankelunas, Vishal D. Murthy, Annie V. Chen, Katie M. Minor, Steven G. Friedenberg, Jonah N. Cullen, Ling T. Guo, James R. Mickelson, and G. Diane Shelton*

2510 **Feline morbillivirus infection associated with fatal encephalitis in a Bengal cat**
*Kara L. D. Dawson, Nicole Wildi, Mauro Cavalli, Dennis Rubbenstroth, Anna Oevermann, and Torsten Seuberlich*

*Respiratory*

2514 **Treatment of recurrent hunting-associated respiratory distress episodes in 2 dogs**
*Renaud Dumont, Chloé Touzet, Eymeric Gomes, and Kevin Le Boedec*

**EQUINE**

Standard Articles

*Endocrinology*

2520 **Short-term effects of canagliflozin on glucose and insulin responses in insulin dysregulated horses: A randomized, placebo-controlled, double-blind, study**
*Sanna Lindåse, Katarina Nostell, Anders Forslund, Peter Bergsten, and Johan Bröjer*

*Gastroenterology*

2529 **Efficacy of the oral supplement, Equine Omega Complete, for the prevention of gastric ulcers and alpha-tocopherol supplementation in horses**
*Elizabeth Williams Louie, Jorge Nieto, Fiona Wensley, Jessica M. Morgan, Carrie J. Finno, and Emily H. Berryhill*

2535 **Right dorsal colitis in horses: A multicenter retrospective study of 35 cases**
*Jordan Flood, David Byrne, Jennifer Bauquier, Gustavo Ferlini Agne, Jessica C. Wise, Carlos E. Medina-Torres, Kelly Wood, Olivia Sullivan, and Allison J. Stewart*

*Nephrology/Urology*

2544 **Effects of phenylbutazone, firocoxib, and dipyrone on the diuretic response to furosemide in horses**
*Julianne M. White, Aimee C. Colbath, and Harold C. Schott II*

*Nutrition*

2552 **The effects of feeding sodium chloride pellets on the gastric mucosa, acid-base, and mineral status in exercising horses**
*Farina Alshut, Monica Venner, Gunilla Martinsson, and Ingrid Vervuert*

*Pharmacology*

2562 **Longitudinal effects of oral administration of antimicrobial drugs on fecal microbiota of horses**
*Diego Gomez, Ramiro Toribio, Benjamin Caddey, Marcio Costa, Stephanie Vijan, and Katarzyna Dembek*

*Respiratory*

2573 **Associations between postrace atrial fibrillation and measures of performance, racing history and airway disease in horses**
*Laura C. Nath, Adrian Elliott, Andre La Gerche, Joe Weir, Grace Forbes, Gijo Thomas, and Samantha Franklin*

**FOOD ANIMAL**

Standard Articles

*Dermatology*

2584 **Prevalence of cutaneous and mucosal lesions in dairy cattle admitted to a Canadian Veterinary Teaching Hospital from 2018 to 2019**
*Eloi Guarnieri, Frédéric Sauvé, Julie Arsenault, and David Francoz*

*Hematology*

2592 **Evaluation of the relationship between ionized and total calcium concentrations in blood during the first week of lactation in dairy cows**
*Imke Cohrs, Sophia Wächter, Lennart Golbeck, and Walter Grünberg*

*Nephrology/Urology*

2603 ***MOCOS*-associated renal syndrome in a Brown Swiss cattle**
*Joana G. P. Jacinto, Leonore Bettina Küchler, Laureen M. Peters, Elke Van der Vekens, Corinne Gurtner, Franz R. Seefried, Mireille Meylan, and Cord Drögemüller*

*Respiratory*

2610 **Differentiating airway inflammation in calves based on cluster analysis of bronchoalveolar lavage fluid cytology**
*Justine Clinquart, Katharina van Leenen, Jade Bokma, and Bart Pardon*

*Theringology*

2623 **Multicenter study of uterine tears and other reproductive complications in periparturient goats presented to veterinary teaching hospitals**
*Lisette M. Coll-Roman, Catalina Cabrera, Ashley R. VanderBroek, Anje G. Bauck, Audrey A. Kelleman, Malgorzata A. Pozor, Jenna W. Stockler, Caitlin Wiley, Clare Scully, Evelyn E. Mackay, Sarah M. Depenbrock, Marie-Eve Fecteau, Michelle Abraham, Laurence Leduc, Charlene V. Noll, Jorge A. Hernandez, and Daniela Luethy*

Case Report

*Hematology*

2631 **A missense variant in *DGKG* as a recessive functional variant for hepatic fibrinogen storage disease in Wagyu cattle**
*Joana G. P. Jacinto, Peter Wohlsein, Irene M. Häfliger, Michael Karl, Michael Pohlers, Lutz Plobner, Walter Grünberg, and Cord Drögemüller*

**ABSTRACTS**

2638 **2023 ACVIM Forum Research Report Program**

### Journal of Veterinary Internal Medicine

ACVIM American College of Veterinary Internal Medicine

Open Access

The Official Journal of the American College of Veterinary Internal Medicine,
the European College of Veterinary Internal Medicine - Companion Animals, the European College of Veterinary Neurology,
and the European College of Equine Internal Medicine

### EDITORIAL BOARD

Kenneth W. Hinchcliff, BVSc, PhD, DACVIM
Editor-in-Chief
The University of Melbourne
Stephen P. DiBartola, DVM, DACVIM
Editor-in-Chief
The Ohio State University
Joshua Stern, DVM, PhD, DACVIM
Associate Editor - Cardiology
University of California, Davis
Jose Novos Matos, DVM, PhD, DECVIM-CA, AFHEA, MRCVS
Associate Editor - Small Animal Internal Medicine, Cardiology
University of Cambridge
Sébastien M. C. Buczinski, DMV, MSc, DACVIM
Consulting Editor for Experimental Design & Statistics
University of Montreal
George Moore, DVM, MS, PhD, DACVIM
Consulting Editor for Experimental Design & Statistics
Purdue University
Kristopher J. Hughes, BVSc (Hons 1), FANZCVS, DECEIM, Associate ACVIM
ECEIM Associate Editor - Large Animal Internal Medicine (Equine)
Charles Sturt University
Linda Kidd, DVM, PhD, DACVIM
Associate Editor - Small Animal Internal Medicine (Infectious Disease)
Linda Kidd Veterinary Internal Medicine Consulting
M. Julia B. Felippe, MV, MSc, PhD, DACVIM
Associate Editor - Large Animal Internal Medicine (Equine)
Cornell University
Shelly L. Vaden, DVM, PhD, DACVIM
Associate Editor - Small Animal Internal Medicine (Nephrology/Urology)
North Carolina State University
Michael Willard, DVM, DACVIM
Associate Editor - Small Animal Internal Medicine
Texas A&M University
Andrea Tipold, DVM, DECVN
ECVN Associate Editor - Neurology
School of Veterinary Medicine Hannover
Robert Rebhun, DVM, PhD, DACVIM
Associate Editor - Hematology/Oncology
University of California, Davis
Eric Zini, PD, PhD, DECVIM-CA
ECVIM-CA Associate Editor - Small Animal Internal Medicine (Endocrinology)
University of Zurich & Istituto Veterinario di Novara & University of Padua
Munashe Chigerwe, BVSc, MPH, PhD, DACVIM
Associate Editor - Large Animal Internal Medicine (Food & Fiber)
University of California, Davis
Lauren Trepanier, DVM, PhD, DACVCP, DACVIM
Associate Editor – Small Animal Internal Medicine (Pharmacology, Pharmacokinetics, Toxicology, and Therapeutic Trials)
University of Wisconsin
Valerie Parker, DVM, DACVIM
Associate Editor - Small Animal Internal Medicine, Nutrition
The Ohio State University
Steven Friedenberg, DVM, PhD, DACVECC
Associate Editor - Small Animal Internal Medicine (Genetics, Genomics)
University of Minnesota
Anthony Mutsaers, DVM, PhD, DACVIM
Associate Editor - Oncology
University of Guelph

**ACVIM Board of Regents Officers**

Jane Sykes, BVSc (Hons), PhD, MBA, DACVIM
Board Chair
Joerg Steiner, DMV, PhD, DACVIM, DECVIM-CA
President
Bill Tyrrell, DVM, DACVIM
Treasurer

**Ex-Officio Member of the ACVIM Board of Regents**

Harold McKenzie, DVM, MS, DACVIM
Immediate Past Board Chair

**The Editorial and ACVIM Offices are located at:**

116 Inveress Drive East, Suite 375, Englewood, CO 80112

**Telephone:** 303-231-9933 or 800-245-9081

**Editorial Email:** JVIM@ACVIM.org.
**ACVIM Email:** ACVIM@ACVIM.org.

The Journal is available online at www.wileyonlinelibrary.com/journal/jvim.

**ACVIM Board of Regents Members**

Sandra D. Taylor, DVM, PhD, DACVIM
Large Animal Internal Medicine At Large
Karyn Harrell, DVM, DACVIM
Small Animal Internal Medicine At Large
Phoebe A. Smith, DVM, DACVIM
Private Practice At Large
Roger A. Hostutler, DVM, MS, DACVIM
President, Small Animal Internal Medicine
Chris Sanchez, DVM, PhD, DACVIM
President, Large Animal Internal Medicine
Steven L. Rosenthal, DVM, DACVIM
President, Cardiology
Joan R. Coates, DVM, MS, DACVIM
President, Neurology
Chad Johannes, DVM, DACVIM
President, Oncology
Shannon Carter, EdD, CAE
Chief Executive Officer

### Journal of Veterinary Internal Medicine

Open Access

American College of **Veterinary** Internal Medicine

The Official Journal of the American College of Veterinary Internal Medicine,
the European College of Veterinary Internal Medicine - Companion Animals, the European College of Veterinary Neurology,
and the European College of Equine Internal Medicine

**Disclaimer**
The Publisher, the American College of Veterinary Internal Medicine and Editors cannot be held responsible for errors or any consequences arising from the use of information contained in this journal; the views and opinions expressed do not necessarily reflect those of the Publisher, the American College of Veterinary Internal Medicine or Editors, neither does the publication of advertisements constitute any endorsement by the Publisher, the American College of Veterinary Internal Medicine or Editors of the products advertised.

**Open Access and Copyright**
Articles published by the *Journal of Veterinary Internal Medicine* are fully open access: immediately freely available to read, download and share. *Journal of Veterinary Internal Medicine* articles are published under the terms of the Creative Commons Attribution Non-Commercial License (CC BY NC), which permits use, distribution and reproduction in any medium, provided the original work is properly cited and is not used for commercial purposes.
Copyright on any research article published in the *Journal of Veterinary Internal Medicine* is retained by the author(s). Authors grant Wiley a license to publish the article and identify itself as the original publisher. Authors also grant any third party the right to use the article freely as long as its integrity is maintained and its original authors, citation details and publisher are identified. The Wiley Open Access Agreement formalizes these and other terms and conditions of publishing research articles.

**Use by commercial "for-profit" organizations**
Use of Wiley Open Access articles for commercial, promotional, or marketing purposes requires further explicit permission from Wiley and will be subject to a fee. All requests to republish the article (in whole or in part) in another work such as a book or journal article (other than normal quotations with an appropriate citation) can be cleared directly from the point of content using the automated RightsLink service. Simply click on the 'Request Permission' link to complete your order.
For other commercial, promotional, or marketing purposes please contact corporatesales@wiley.com. Commercial purposes include: Copying or downloading of articles, or linking to such articles for further redistribution, sale or licensing; Copying, downloading or posting by a site or service that incorporates advertising with such content; The inclusion or incorporation of article content in other works or services (for example, a compilation educational purposes; Use for the purposes of monetary reward by means of sale, resale, licence, loan, transfer or other form of commercial exploitation such as marketing products; Print reprints/eprints of Wiley Open Access articles.

*JOURNAL OF VETERINARY INTERNAL MEDICINE* (ISSN 1939-1676 [online]) is published bimonthly on behalf of the American College of Veterinary Internal Medicine by Wiley Periodicals LLC, 111 River St., Hoboken, NJ 07030-5774.

**Publisher**
*Journal of Veterinary Internal Medicine* is published by Wiley Periodicals LLC, 350 Main St., Malden, MA 02148-5020

**Production Editor:** Deepika Prakash (email: JVIM@wiley.com).

**Commercial Reprints:** Helane Daniels (email: hdaniels@wiley.com)

**Abstracting and Indexing Services**
The Journal is indexed by Science Citation Index, and MEDLINE. For a complete list of A&I Services visit: www.wilevonlinelibrarv.com/iournal/jvim

ISSN 1939-1676 (Online)

For submission instructions, and other information visit: www.wileyonlinelibrary.com/journal/jvim

**Manuscript Review**
All manuscripts are reviewed by experts in the field who advise the editors of the manuscript's scientific quality. Decisions regarding publication are made by the Co-Editors-in-Chief acting on the advice of reviewers and Associate Editors.

Wiley's Corporate Citizenship initiative seeks to address the environmental, social, economic, and ethical challenges faced in our business and which are important to our diverse stakeholder groups. Since launching the initiative, we have focused on sharing our content with those in need, enhancing community philanthropy, reducing our carbon impact, creating global guidelines and best practices for paper use, establishing a vendor code of ethics, and engaging our colleagues and other stakeholders in our efforts.

Follow our progress at www.wiley.com/go/citizenship
